# Method for the Mixing Design and Physical Characterization of Air-Foamed Lightweight Clay Concrete: A Response to the Issue of Recycling Dredged Sediments

**DOI:** 10.3390/ma17246248

**Published:** 2024-12-20

**Authors:** Agnès Zambon, Zoubir Mehdi Sbartaï, Nadia Sayouri

**Affiliations:** 1 Laboratoire de Génie Civil et Géo-Environnement, University Lille, IMT Nord Europe University Artois, Yncrea Hauts de France, ULR 4515-LGCgE, 59000 Lille, France; 2 I2M (Institut de Mécanique et d’Ingénierie), UMR 5295, CNRS, University of Bordeaux, 33400 Talence, France; zoubir-mehdi.sbartai@u-bordeaux.fr (Z.M.S.); nadia.saiyouri@u-bordeaux.fr (N.S.)

**Keywords:** sediments, dredging, recycling, air foam, lightweight concrete, compressive strength, workability, porosity, X-ray tomography

## Abstract

From both economic and environmental points of view, the reuse of dredged sediments in the direct onsite casting of concrete represents a promising method for replacing sand. The aim of this study was to develop a cementitious material that (i) reuses the thin particles of sediments; (ii) has a low density due to the incorporation of air foam in the material; and (iii) achieves a minimum mechanical strength of 0.5 MPa for embankment applications. This study focused on the characterization of a non-standard “concrete”, which is a mixture of a synthetic soil (80% montmorillonite and 20% calibrated sand) and cement. To reduce its density, air foam was incorporated into the material during the manufacturing process (air-foamed lightweight clay concrete—AFLCC). The study results highlight that a density around 1.2 (unit: g/cm^3^/1 g/cm^3^) can be obtained. This density reduction can be obtained with a certain degree of workability when the material is in a fresh state. To obtain this workability, a certain amount of water must be added; however, the addition of water has a significant impact on the compressive strength of the AFLCC. As such, a mathematical equation correlating the compressive strength, the density, and the percentage of cement is proposed in this study. The mechanical strength results of the AFLCC at different times, in conjunction with the Vicat results, show that the porosity created by the air foam has the effect of slowing down the hydration mechanism of the cement. The porosities obtained are consistent with the density results. The characteristic radii indicate large pore sizes for formulations with low fluidity in the fresh state when air bubbles are incorporated.

## 1. Introduction

Every year, about 50 million cubic meters of sediments are dredged in France [1] Until the 1990s, the dredged sediments were released somewhere else in the sea/ocean. However, this method poses a threat to the littoral ecosystem. Thus, in some cases, dredged sediments must be dumped out at sea, according to legislative recommendations [2] In this case, sediments are considered by the legislation to be waste. Most of the valorization solutions related to dumped sediments are expensive and consume large amounts of energy. Directly reusing dumped sediments as raw material in the process of concrete casting on dredging sites represents an interesting economical and eco-friendly solution to this problem [3,4,5,6,7,8,9]. However, the thin particles of sediments (a fraction under 2 µm, which corresponds to clay) are not widely reused nowadays. As clay has physical and chemical properties with higher variability than sand, the use of clay in cementitious materials is particularly complicated. Indeed, the hydrated phyllosilicates of the aluminum in the clay make it prone to react with moisture and confer it with swelling and low-strength characteristics. Moreover, it gives the clay the capacity to retain polluting agents, such as heavy metals and hydrocarbon [10,11]. Several studies highlight these specific features and the difficulties of using clay in building applications; these studies focus on improving the physical characteristics of clay using cement treatments [12,13,14], lime sludge and cement treatments [15,16], fly ash treatments [17], or with the incorporation of polypropylene fibers [18].

It is challenging to optimize the valuable proportion of clay by achieving the total substitution of fine aggregates in the cementitious materials with the thin particles of sediments. The reviewed literature generally focuses on applying this material for geotechnical infrastructure, such as embankments [2,19]. The main characteristic of embankment materials is their low density. Nowadays, many manufacturing methods are used to reduce the density of cementitious materials in order to use them as mass void fillers or for tunnel stabilization or pavement bases, etc. [19,20,21]. Of particular interest among lightweight cementitious materials is air-foam concrete, which is still under development. The most common on-site manufacturing method used for industrial applications involves incorporating air foam into the concrete during the mixing procedure; the air foam (air bubbles based on animal protein) is generated beforehand using an air compressor. The material developed in the present study was produced using this method. It is classified as a lightweight foamed concrete (LWFC) or as a foamed lightweight soil (FLS). In this paper, the material developed is referred to as AFLCC, or “air-foam lightweight clay concrete”. To date, air-foam concrete with sediments has been used in several construction sites in Korea, Thailand, and Japan, representing more than 300,000 m^3^ of concrete [21]. It was used to construct the lining of the Yume–Shima tunnel which connects Yumeshima Island and Sakishima in Osaka Bay [22,23]. On this construction site, the use of air-foam concrete allowed the load applied to the tunnel to be reduced. During this process, the concrete is manufactured and placed directly into the water from a vessel. The density and the target strength are 1.12 (unit: g/cm^3^/1 g/cm^3^) and 0.6 MPa, respectively. Foam concrete based on marine-dredged sediments has also been used as a filling material in Kumamoto harbor in Japan [24]. The use of air-foam concrete with sediments reduces the thrust of the structure on the ground and allows for the reuse of sediments dredged in the harbor. The manufacturing process takes place directly at the dredging site. The sediments used in this project have a liquidity limit of 63.8%. The target density and mechanical strength of the sediment-based foam concrete are, respectively, 1.1 and 0.2 MPa [24]. In support of the implementation of these projects, several studies for the reuse of sediments (or clay) in air-foamed concrete have been carried out in recent years [19,25,26]. These studies generally focus on using the material as backfill or embankment. S. Chaiyaput et al. found that the compressive strength and durability of FLS decreases with the percentage of soil in the formulation, but it is applicable in subgrade engineering (>0.3 MPa) and represents an environmentally sustainable alternative in embankment engineering [26]. A recent study [27] concluded that air-foam lightweight cemented-clay materials are suitable for use as a backfill material for constructing embankments on a soft clay foundation. On the basis that foam concrete has high porosity and high water-permeability, a recent study [28] investigated the durability of foam concrete. Another study [29] showed that foam concrete has good freeze–thaw cycle resistance but exhibits poor carbonation resistance and chloride resistance.

With regard to the mixing design methods, a study performed in Thailand by Horpibulsuk [23] set up a design method with a liquid limit for the soil and water content. It is referred as w/w_L_ and corresponds to the following ratio {sediment water content/liquid limit of sediment}. The fresh density of concrete as a function of this presented a transition zone. This transition zone must be exceeded in order to significantly reduce the material’s density. This transition zone differs according to the type of sediment, but the study concludes that the optimum value of the w/w_L_ ratio that is applicable to all types of soil is about 1.9. A study performed by Lee [30] highlighted the fact that air-foam concrete with sediments exhibited ductile or brittle mechanical behaviors depending on the formulation parameters. For a fixed quantity of cement, a fixed quantity of sand, and a density that varies based on the addition of more or less foam, the behavior of the material changes from brittle to ductile.

This study was carried out with the following goals:

(1) To understand and explain how the incorporation of air foam influences the physical properties (density, spreading, and mechanical strength) of the developed ALFCC.

(2) To determine how the mixing design parameters, such as the water and cement fraction, influence the incorporation of the air foam and thus, how they can change the physical properties (density, spreading, and mechanical strength) of the ALFCC.

(3) To ensure that the developed material, ALFCC, can be used for the target application in embankments (requiring a density under 1.2 (unit: g/cm^3^/1 g/cm^3^) and a spreading diameter greater than 40 cm).

A mixing design method is presented in this study. The developed method allows us to target a given density and mechanical strength in the ALFCC. It is based on a mathematical equation which correlates compressive strength and density. This also enables the fixing of the mix design according to the application. Mechanical and physical properties, such as the mechanical strength, elasticity modulus, and density, were measured, and the results are presented below.

## 2. Materials and Methods

### 2.1. Artificial Soil

**Figure 1:**
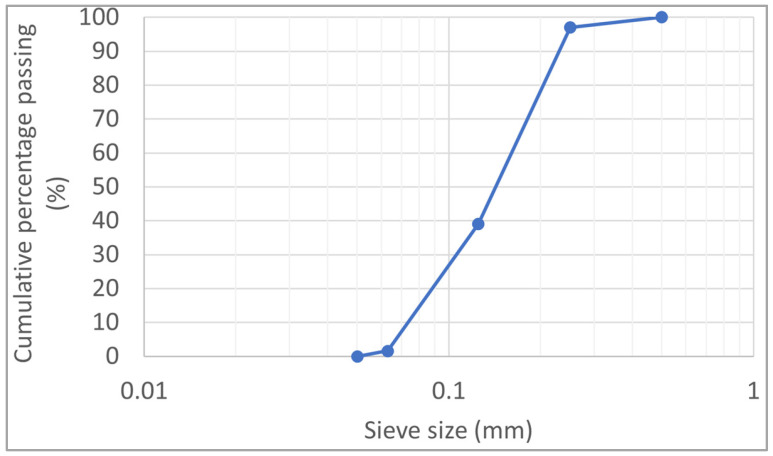
Grading curve of the sand.

To prevent variability in the clay sediment between samples, an artificial soil was used in this study. This artificial soil comprised 80% calcic bentonite and 10% calibrated sand (maximum diameter of 0.125 mm). Figure 1 presents the grading curve of the sand.

Calcic bentonite was chosen because it is the material that is most commonly found in a natural state. The proportions of the artificial soil were fixed to obtain a liquid limit amount of about 90%. This value was set in relation to the values found in the literature and following measurements of liquidity limits carried out on sediment samples in the Arcachon basin.

To know the proportion of sand/clay needed to obtain a liquidity limit of 90%, a preliminary study was carried out; the liquidity limit was measured on several soils with different sand/clay proportions.

The characteristics of the bentonite and sand used are presented Table 1 and Table 2, respectively. 

### 2.2. Cement

The cement used was CEMI 52.5 N CE PM CP2 NF from the manufacturer CALCIA (Bussac, France). The mechanical and physical characteristics and components of the cement are presented in Table 3 and Table 4.

### 2.3. Air Foam

The air-foam generator used in this study was manufactured by the manufacturer ProPump Engineering (Dartford, UK). The air foam was made upstream and then incorporated into the mix “artificial soil + water + cement”. The air foam is produced as follows: The air-foam generator is connected to an air compressor, which supplies an airflow rate equal to 7 × 10^−3^ m^3^/s with an air-pressure of 0.7 MPa. It is also connected to a bottle of liquid (supplied by ProPump). When the air compressor is opened, the liquid is pumped into the generator and emulsified. The foam then exits through another hose in the generator and is collected in a tray. The required quantity is then taken and added to the mortar mix in the mixer.

The liquid is eco-friendly, as it is made from animal proteins (hooves, horns) [33]. The liquid used in this study was provided by ProPump. It was diluted with a multiplying factor of 25. Under these conditions, the air-foam density is about 0.05. 

### 2.4. Mixing Design Method

The mixing design method involves four components: water, cement, dry artificial soil (bentonite + sand), and air foam. The proportions of these components were fixed with reference to three parameters. These parameters are defined according to two concerns:

1. Treatment with a hydraulic binder (cement) of soil with some water content.
2. Reducing the density by incorporating air foam.

The first mixing design parameter sets the proportions of the water and soil. They are fixed as a water content, that is, a mass ratio, using the following formula:

(1) $$w(\%)=\frac{\mathrm{mass}\;\mathrm{of}\;\mathrm{water}}{\mathrm{mass}\;\mathrm{of}\;\mathrm{dry}\;\mathrm{soil}}\times 100$$

To account for the swelling of the soil in contact with water, the water content w is divided by the liquid limit (w_L_). This ratio w/w_L_ is defined and used as a mixing design parameter by Horpibulsuk [23]. This ratio (w/w_L_) is the first mixing design parameter, which fixes the quantity of water and the proportion of dry soil. In this study, tap water is added to obtain the fixed water content. Practically, on site, the quantity of water is a parameter that is known and imposed as input data. In this study, the water quantity varies. Thus, cases with different water-content levels of sediment can be studied.

The second mixing design parameter determines the proportion of cement. It is fixed as a mass compared to the mass of the air-foam lightweight concrete (AFLCC). It is noted that the mass of the air-foam lightweight concrete corresponds to the mass of the mix “soil + water + cement” because the mass of the air foam is neglected. This parameter is referred to as “C” and corresponds to the percentage by weight of the cement (compared to the total weight of the concrete).

The third mixing design parameter sets the quantity of the air foam. It is fixed as a volume as compared to the volume of the mix “soil + water + cement”. It is a percentage and is referred to as “M”.

The mixing design method has the following steps.

- The first step corresponds to calculating the mass of cement from the mass of the AFLCC.
- Then, the masses of the soil and the water are calculated from the value of the w/w_L_ ratio.
- The mixing design method requires measuring the density of the “soil + water + cement” mix for each mix to calculate the volume of “soil + water + cement” in order to calculate the mass of the air foam.

### 2.5. Experimental Program

The percentage of cement by weight was fixed to 4%, 8%, 12%, 15%, and 18%. For each percentage of cement, several w/w_L_ ratios were tested to study various mixes with different water contents. For each w/w_L_ ratio, several percentages of air foam were tested (20%, 30%, and 50%).

It should be noted that density was measured for all the tested mixes (percentage of cement of C = 4%, C = 8%, C = 12%, C = 15%, and C = 18%). Regarding the mechanical strength and elastic modulus, mechanical tests were not conducted for all of the mixes. Indeed, given the results obtained for the first mix design tested with C = 4% and C = 8% and with some ratio of w/w_L_, mechanical tests were not performed for all other values. This is because the target strength was not reached. That is why the percentage of cement was increased to C = 12%, C = 15%, and C = 18%.

### 2.6. Mixing Procedure

**Figure 2:**
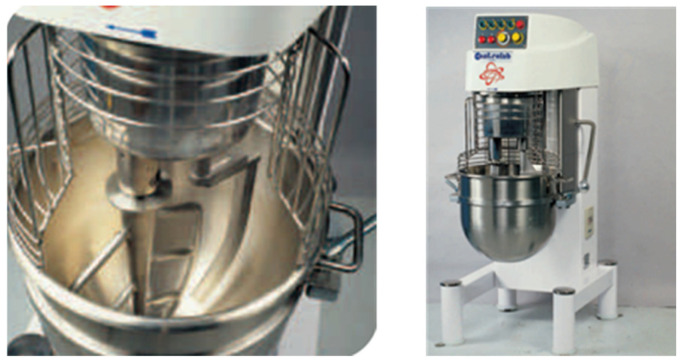
Planetary rotation mixer.

The components were mixed in a planetary rotation mixer (Figure 2) according to the following steps.

**Figure 3:**
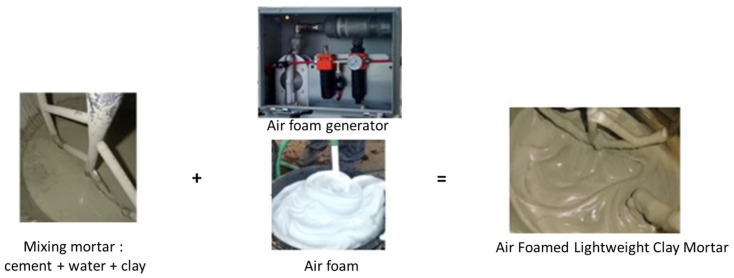
Steps for mixing mortar and air foam.

Soil and water were first mixed for 30 min with a velocity of 30 rpm. The cement was added and mixed for 10 min. Finally, air foam was added and mixed for 1 min (Figure 3).

**Figure 4:**
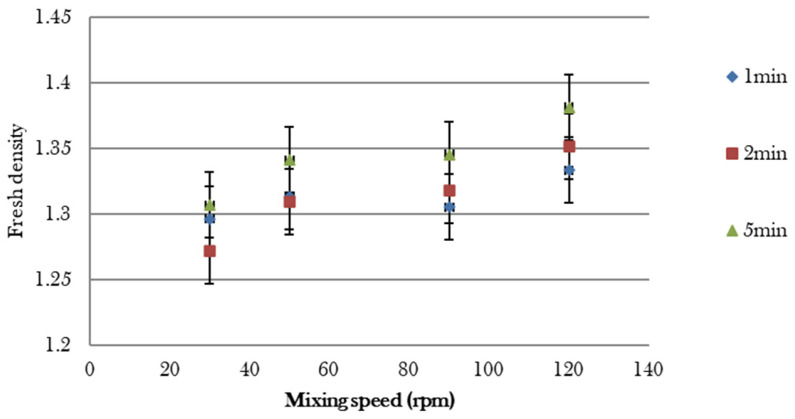
Density according to the mixing time and mixing speed.

A preliminary study was required to determine the time and speed at which the foam was incorporated into the mixture. The incorporation of the foam into the mixture presents the problem of the bursting of air bubbles, which is influenced by the mixing energy. In this study, this effect was minimized while ensuring a homogeneous mix. To assess the optimum mixing combination (time and speed), 12 batches were produced, corresponding to a combination of 4 mixing speeds (30 rpm, 50 rpm, 90 rpm, and 120 rpm for the scraper arm) and 3 mixing times (1 min, 2 min, and 5 min). Three fresh density measurements were taken to assess the variability of the measurement. Figure 4 indicates that density increases with the mixing time and mixing speed. The homogenization of the mixture is visually improved for longer mixing times but is at the correct level with a time of 1 min and a mixing speed of 30 rpm. The time and speed for mixing the foam are set at 1 min and 30 rpm, respectively.

Before pouring the fresh concrete, the molds were oiled to facilitate demolding. No process of implementation was used because, for the target application, the embankment materials require high-flowability spreading. Regarding the curing conditions, for the landing phenomena of cracking and shrinkage, the molds were covered before demolding. The samples were demolded after 5 days. This time period was chosen to ensure that the samples were properly demolded for all mixes. After being demolded, the samples were put in a tank with a fixed humidity equal to 90% and a fixed temperature equal to 20 °C.

### 2.7. Experimental Tests

#### 2.7.1. Evaluation of Fresh Properties

The workability of the fresh concrete was studied by measuring the flowability with Abram’s cone in accordance with the EN 12350-8 standard [34]. The spreading was measured a few minutes after mixing the concrete, which corresponded to about fifteen minutes after casting. Spreading was measured on several batches of the same mix in order to estimate the variability of the results.

#### 2.7.2. Fresh Density

The density of the fresh concrete was measured using a Baroïd scale. The value of one mix corresponds to an average of three measures. The Baroïd scale is normally used for sludge. It was used to measure density in the present study because it is suitable for measuring densities between 0.9 and 2.8, which are the values under consideration in this study. The scale is robust enough to be used on construction sites.

It is measured in relation to the specific weight of water using the following formula:

$$d=\frac{mv}{{mv}_{water}}$$

with

d; density of the fresh concrete (no unit: g/cm^3^/1 g/cm^3^).

mv; the specific weight measured with the Baroïd scale.

mv_water_; the specific weight of water equal to 1 g/cm^3^.

#### 2.7.3. Compressive Test

**Figure 5:**
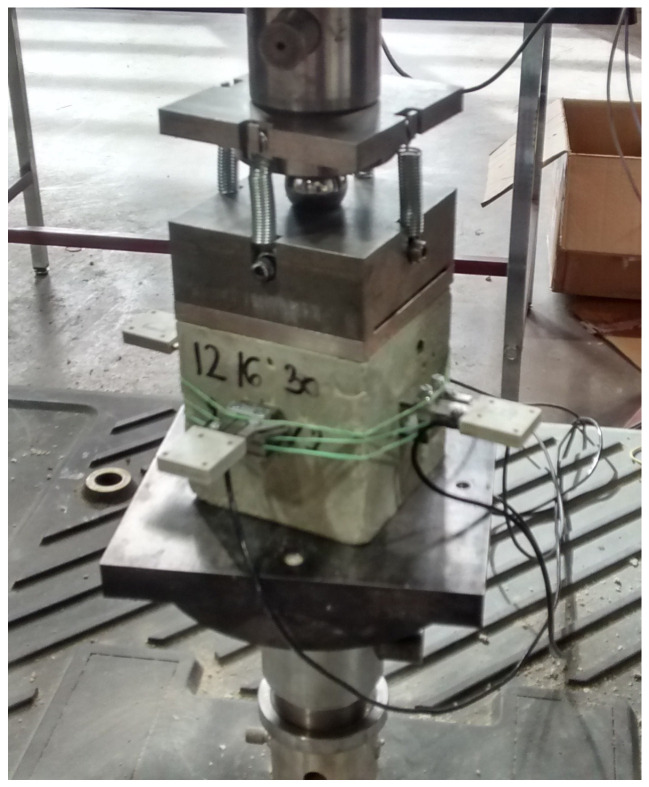
Specimen under compressive test conditions and equipped with longitudinal strain sensors.

The compressive test was conducted on three cubic samples of 100 × 100 × 100 mm^3^ at 28 days for each mix. Some mixes were also tested at 7, 14, and 90 days to study the evolution of the compressive strength over time. The compressive tests were carried out using an electromechanical machine with a capacity of 50 kN. The loading of the concrete was achieved with a steel plate at a constant rate of 0.0015 mm/s by means of jack displacement. The specimens were equipped with four sensors (HBM) to measure the longitudinal strain (Figure 5). HBM corresponded to blades that were stuck in the material. The mechanical movement of the blades was converted into an electrical signal to measure the variation in the distance between the blades (ΔL) during the test. These data were used to calculate the longitudinal strain during the test according to the following formula:

(2) $$\varepsilon_{l}=\frac{∆L}{L}$$

Strain and strength were measured every 0.02 s. Based on these tests, curves of stress according to longitudinal strain were plotted for each sample. For each mix, the elastic modulus and compressive strength (average of all samples) were calculated from these curves as follows:

An elastic field is defined from 10% up to 30% of the maximum stress. The elastic modulus corresponds to the slope of the linear regression of the stress–deformation curve. The compressive strength corresponds to the maximum force divided by the sample section in MPa.

#### 2.7.4. Vicat Test

The Vicat test defines the beginning of the setting time and the end of the setting time of a cementitious material. It was carried out in accordance with the standard NF EN 196-1 [35] for the mixes, corresponding to

- C = 18%, w/w_L_ = 2, M = 0%
- C = 18%, w/w_L_ = 2, M = 50%.

#### 2.7.5. X-Ray Tomography

The 2D images (corresponding to one layer of the sample) were processed using Fiji software (ImageJ 1.51s) in order to quantify the proportion of pores and to determine the granulometric distribution of pore size. The pores studied mainly corresponded to the air-foam porosity. However, due to the release of gas from the hydration mechanisms and clay shrinkage, cementitious matrix porosity is taken into account; this is because it corresponds to the same order of magnitude as certain pores in the foam, and it is difficult to differentiate and remove it with the 2D image treatments.

The various processing treatments used to obtain this information are detailed in Appendix B. These treatments were defined for each formulation in order to visually select only the porosity of the foam. The porosity due to release of gas from the hydration mechanisms and clay shrinkage can be visualized and differentiated from that of the foam by using the images of the M = 0% reference formulation.

## 3. Results and Discussion

### 3.1. Fresh Concrete: Workability and Density

**Figure 6:**
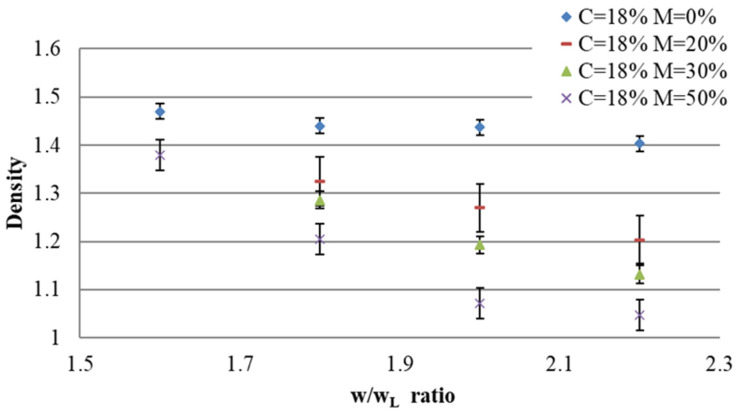
Density according to w/w_L_ for C = 18%.

Figure 6 presents the density of the fresh concrete according to the w/w_L_ ratio for various percentages of foam for the mixes with cement C = 18%. This figure was analyzed in conjunction with the workability results in order to study the changes in density with respect to workability. The aim is to determine the stability of the air foam in regards to these two parameters. For low values of w/w_L_ up to 1.6, the density of the fresh concrete does not vary with percentages of air foam. The low workability of the “soil + water + cement” mix caused the collapse of the air foam during the mixing. The energy required to incorporate the air bubbles into the mortar is probably greater than the energy required to stabilize the foam bubbles. By combining the density results with the spreading test for the fresh state, a link between these two parameters is established. For low values of w/w_L_ up to 1.6, the spreading diameter is lower than 30 cm. Thus, for this value of diameter spreading, all air-foam bubbles collapse, and it is impossible to decrease the density in this part of the graph (w/w_L_ < 1.6).

Up to a w/w_L_ ratio value of about 2, the density of the fresh material decreases and is different for the different air-foam percentages. Thus, the densities according to various percentages of air foam are different. These observations can be explained by the fact that the phenomenon of the collapse of the air foam becomes less and less significant. Indeed, for this higher value of the w/w_L_ ratio, the workability of the “soil + cement + water” mix is higher and thus, air-foam bubbles are introduced more thoroughly into the mix. As shown in Appendix A, for this value of w/w_L_, the spreading diameter is equal to 35 cm. Thus, the addition of air foam decreases the density.

It is important to note that air foam has an impact on the workability of the material. For a particular percentage of cement and fixed w/w_L_ ratio, the workability of the fresh material increases with the percentage of air foam. The density and workability values of the tested mix design are well matched to the characteristics expected for the target application, i.e., a density under 2 and a spreading diameter above 30 cm. For example, for a mix with a percentage of cement equal to 18%, a w/w_L_ ratio equal to 2, and with 50% air foam, the spreading diameter and densities are 47.25 cm and 1.07, respectively.

Based on a w/w_L_ ratio equal to 2, the densities of the different mixes decreased. The densities are still different depending on the air-foam percentage. However, unlike the previous phase, the densities decrease in a similar way regardless of the percentage of air foam. In this phase, the collapse of the air foam is minimized and the reduction in the density is optimized. At a w/w_L_ ratio of 2, workability is at its optimum value for the incorporation of air foam to reduce the density of mortar.

The density and workability values of the tested mix design are well matched to the characteristics required for the target application, as shown in the previous phase. For example, for a mix with a w/w_L_ ratio equal to 2.2 and with 20% of air foam, the spreading diameter is equal to 80 cm. According to the EN 12350-8 standard [34] for self-compacting concrete, the material used for this mix is classed as SF3.

For a w/w_L_ ratio equal to 1.6, the density is optimized and decreases. This value is referred to as “k”. It is defined as an optimum value of the w/w_L_ ratio. The value is specific to this percentage of cement. Producing cement involves mechanisms of hydration, which require water. Increasing the amount of cement consumes more water, reduces workability, and thus causes the air foam to collapse. Therefore, the same trends are observed for C = 4%, C = 8%, C = 12%, and C = 15% for w/w_L_ ratios lower than for C = 18% (all density results are presented in Appendix A). For each percentage of cement, a specific value of the parameter “k” (optimum value of the w/w_L_ ratio) is defined.

**Figure 7:**
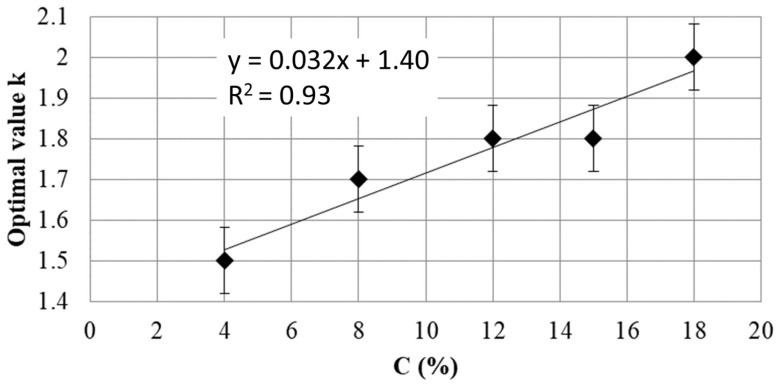
Relationship between k, the optimal value of w/w_L_, and the cement percentage C.

Figure 7 presents the relationship between the optimal value of w/w_L_ (k) and the percentage of cement. A linear regression with a determination coefficient equal to 0.93 is set up between k and the cement percentage C. For any w/w_L_ ratio, it is possible to extract a percentage of cement. Thus, it is possible to determine based on the liquidity limit of the clay the percentage of cement that should be used.

### 3.2. Mechanical Performance of the Hardened Material

**Figure 8:**
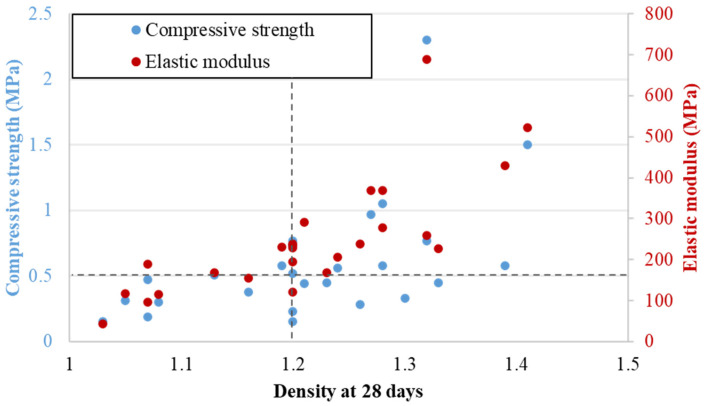
Experimental results for the density, mechanical strength, and elastic modulus of each mix design.

Figure 8 presents the mechanical strength and elastic modulus according to density for all tested mixes. For most of the tested mixes, the obtained compressive strength and density values comply with the specification of 0.5 MPa strength and density lower than 1.2, which are the minimum and maximum targets, respectively. They are obtained for an acceptable percentage of the cement according to the target application (lower than 18%). Mix designs that do not comply with this specification are still interesting in the context of this study, allowing us to propose a mix design methodology for other applications.

**Figure 9:**
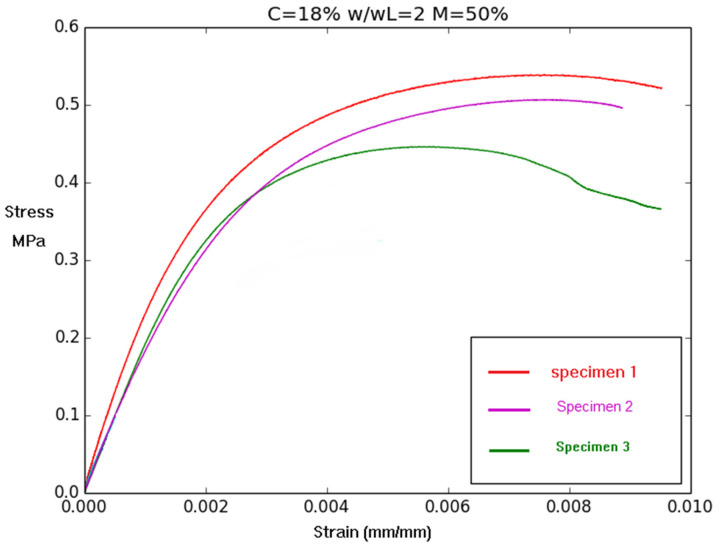
Relationship between stress and strain for each sample for a mix design of C = 18%, w/w_L_ = 2, M = 50%.

For each mix, the coefficients of variation between samples for the compressive strength and elastic modulus are, on average, equal to 0.1%. This low dispersion rate indicates the reliability of results. The elastic modulus and mechanical strength have almost the same variation as a function of density. The variation according to density is described in more detail in Section 3.3. The maximum compressive strength is equal to 2.42 MPa. It is obtained for the mix design with a percentage of cement C = 18%, a ratio w/w_L_ = 1.8, and a percentage of air foam M = 20%. The corresponding density is equal to 1.44, and the corresponding elastic modulus is equal to 688 MPa. It is also the highest value for the elastic modulus among the tested mixes. Typical stress curves according to the longitudinal strain were drawn for each sample. Figure 9 presents the superposition of the curves for all samples for the mix with C = 18%, w/w_L_ = 2, and M = 50% to give an example of the mechanical behavior. For each mix design, the same behavior is observed.

As shown in Figure 9, at first, stress increases linearly with longitudinal strain (up to a strain value of 0.002). This corresponds to the elastic range characterized by the elastic modulus. Then, the increase in stress according to strain is lower. Figure 9 indicates that this phase ranges from 0.2% up to 0.5 or 0.8%, depending on the tested sample. The highest stress defines the compressive strength (referred to as f_c_).

Regarding compressive strength, the decrease in stress indicates the propagation of cracks. At the point of failure of the material, the strain is about 1.0%, which is significantly higher than the values recorded for ordinary concrete, for which the strain level at failure is about 0.4% [36]. This significant strain is a characteristic mechanical behavior of fine soil-based cementitious materials [37] and of air-foam concrete [27].

**Figure 10:**
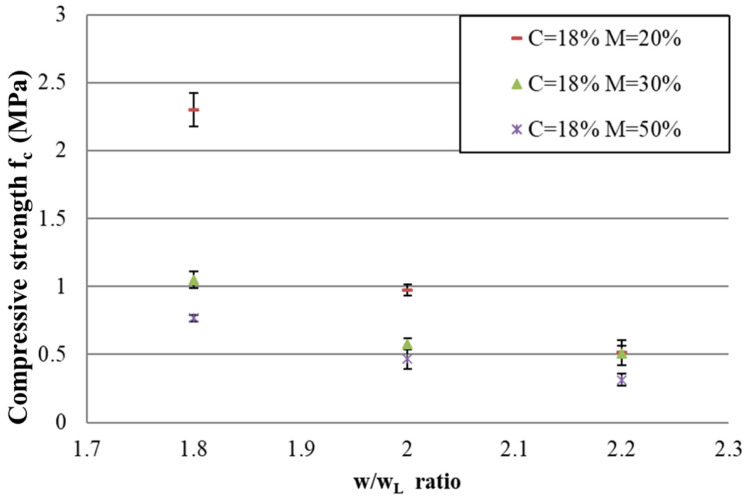
For C = 18%, the relationship between compressive strength fc and w/w_L_ for each percentage of air foam M.

**Figure 11:**
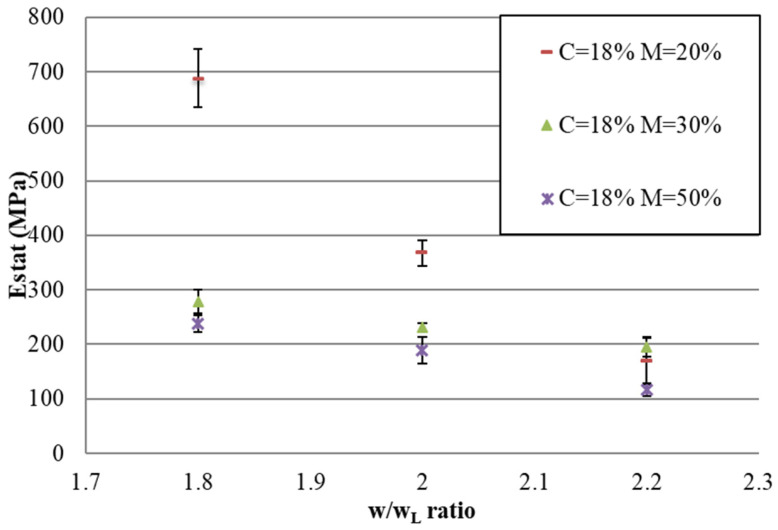
For C = 18%, the relationship between E_stat_ and the w/w_L_ ratio for each percentage of air foam.

Figure 10 and Figure 11 show the compressive strength and elastic modulus for C = 18%, respectively. For other percentages of cement, the compressive strength and elastic modulus are presented in Appendix A, and the same conclusions as presented for C = 18% are drawn.

Results in Figure 10 and Figure 11 show that the compressive strength (as well as the elastic modulus) decreases with the w/w_L_ ratio. This decrease, which is observed for each mix with a different percentage of air foam, can be explained by the addition of water and by the fact that increased water content increases workability; this makes the introduction of air foam into the concrete mix easier, which makes the material more porous.

For mixes with different percentages of air foam, the compressive strength and elastic modulus are more significantly different for low w/w_L_ ratios than for high w/w_L_ ratios. These results are quite unexpected because, as explained for the density results, for low w/w_L_ ratios, air-foam bubbles are not thoroughly incorporated into the mix in the fresh state and collapse during mixing. Thus, the air-foam concrete for low w/w_L_ ratios is quite comparable. The porosity for mix designs with different percentages of air for low w/w_L_ ratios is also quite comparable. This means that, up to a certain w/w_L_ ratio, the porosity due to free water decreases the compressive strength more than the air foam. That is to say, up to a certain w/w_L_ ratio, the compressive strength and elastic modulus are much more heavily influenced by the addition of water than by the air foam. It is therefore beneficial to choose a lower w/w_L_ ratio in order to reduce the density by adding much more air foam without significantly decreasing the mechanical performance.

### 3.3. Variation in Compressive Strength at Different Curing Times

**Figure 12:**
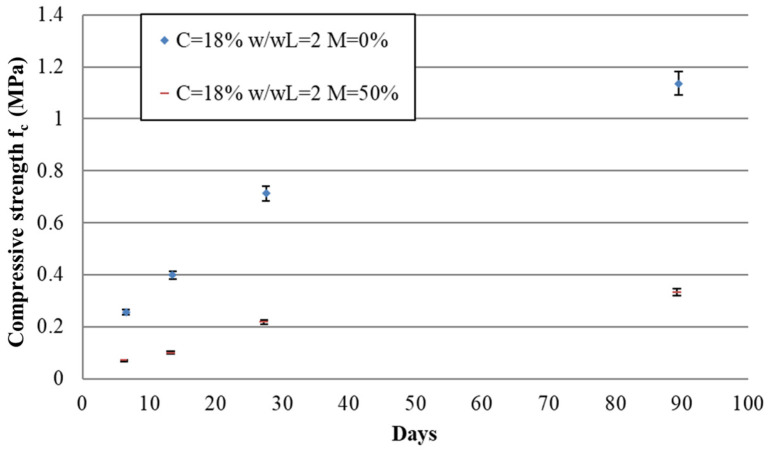
Compressive strength at 7, 14, 28, and 90 days for the four mix designs.

The mechanical tests were carried out at different curing times in order to evaluate the evolution of the compressive strength of the AFLC over time. The tests were carried out at 7, 14, 28, and 90 days. The mechanical strength as a function of time for the tested mixes is presented in Figure 12.

Between 7 days and 28 days, the increase in mechanical strength was almost linear for all mixes. For the mix design with M = 0%, the increase was similar. For the mix design with M = 50%, this increase was also similar but lower than that with M = 0%. This difference in the strength gain between M = 0% and M = 50% may be due to the presence of air-foam-related porosity in the hardened state, which decreases the material’s compressive strength. It may be also increased by a slowing down of the hydration mechanisms caused by the presence of air foam. This effect was investigated using the Vicat test for the early stages (from the first hours) of the cement hydration mechanisms; it can be attributed to a slowing down of the diffusion of ions in the solution, which participate in hydration mechanisms [38,39].

Between 28 days and 90 days, a slight difference in the strength increase between M = 0% and M = 50% was observed. The increase in strength between 28 days and 90 days is also related to the diffusion of ions in the cement matrix and would therefore also be influenced by the presence of air foam, explaining the difference between the mix design with M = 0% and the one with M = 50%.

Between 28 days and 90 days, the increase in mechanical strength is, on average, about 75% for the four tested mixes. This value is clearly higher than that of conventional concrete, which is around 25%. This increase can be attributed to the extension of the hydration mechanisms due to the water absorbed by the clay, which acts as a water reservoir and allows the hydration reactions to continue [7].

### 3.4. Predictive Mathematical Model of Mechanical Properties

**Figure 13:**
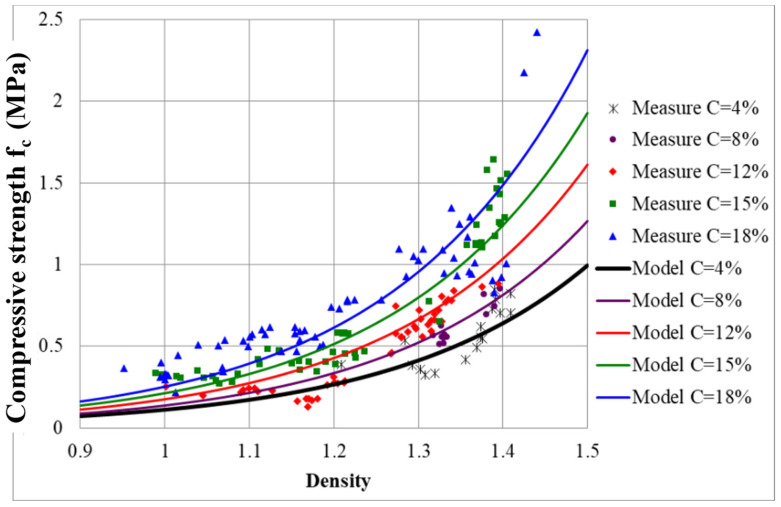
Relationship between compressive strength, density, and cement.

Using experimental data, a predictive mathematical model of mechanical properties in relation to the density and percentage of cement is established. In order to set up this model, experimental results for all mixes of density and of compressive strength were plotted as shown in Figure 13. This figure shows that, for a fixed density, increasing the percentage of cement increases the compressive strength. It appears that a relation between **f_c_** and density (**d**) can be proposed for each percentage of cement (**C**). The compressive strength is correlated to the density at 28 days and to the percentage of cement with the following equation:

(3) $$f_{c}=a_{0\;}{\cdot \;e}^{a_{1}\;\cdot \;\;d}\cdot \;e^{a_{2}\;\cdot \;\;C},$$

where a_0_, a_1_, and a_2_ are equal to 1.05 × 10^−3^, 4.41, and 6.01 × 10^−2^, respectively, and d and c correspond to the density and proportion of cement, respectively.

**Figure 14:**
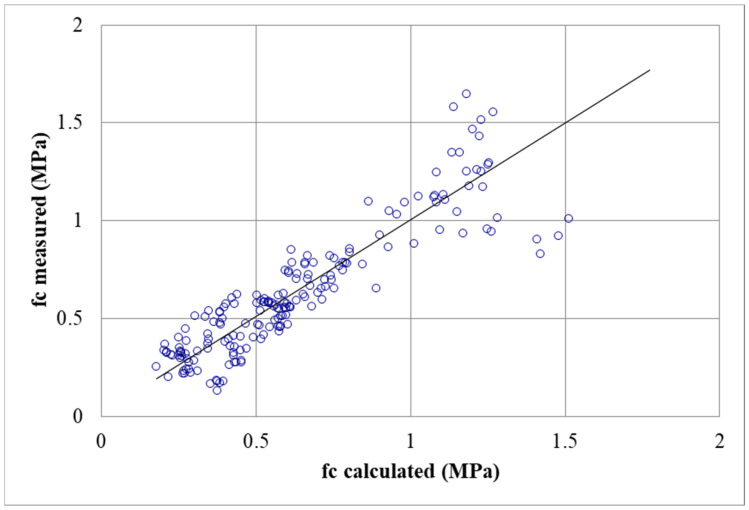
Measured compressive strength with respect to the calculated strength.

The model curves plotted for each percentage of cement (Figure 14) are strongly correlated to the compressive strength, with a correlation coefficient equal to 0.81. This model makes it possible to fix an optimized mix design that corresponds (for density, compressive strength, and percentage of cement) to the chosen value. For the required density and compressive strength, depending on water content and the liquidity limit of the sediment, it is possible to fix the corresponding mix design (percentage of cement and percentage of air foam). For a fixed percentage of cement, there is a range of possible combinations for density and compressive strength.

### 3.5. X-Ray Tomography

**Figure 15:**
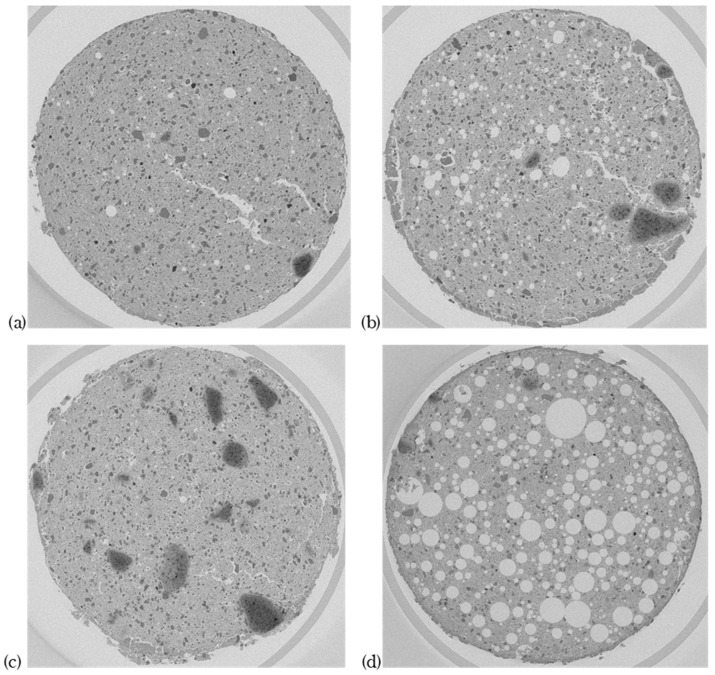
Raw images from X-ray tomography for the mix designs C = 12% w/w_L_ = 1.6 M = 0% (**a**), C = 12% w/w_L_ = 1.6 M = 50% (**b**), C = 18% w/w_L_ = 2 M = 0% (**c**), and C = 18% w/w_L_ = 2 M = 50% (**d**).

Figure 15 presents raw images from X-ray tomography of the following mix designs:

- C = 12%, w/w_L_ = 1.6, M = 0% (a),
- C = 12%, w/w_L_ = 1.6, M = 50% (b),
- C = 18%, w/w_L_ = 2, M = 0% (c),
- C = 18%, w/w_L_ = 2, M = 50% (d).

The images of the formulations where M = 0% exhibit spherical coarse pores, which are the result of gaseous outgassing during cement hydration.

The images of the formulations where M = 0% show internal cracks, more than the formulations with M = 50%. The presence of foam reduces the internal tension exerted by the shrinkage of the clay. Cracks are also more pronounced for C = 12% than for C = 18%. This is explained by the lower cement content. The difference in the w/w_L_ ratio between the two formulations, with cement percentages of C = 12% and C = 18%, should have an impact on the appearance of cracks. However, the tendency is that the higher the w/w_L_ ratio, the more likely it is that cracks will form. This is because the clay will swell more and therefore shrink more. This effect is not observed, and we can consider that the impact of the quantity of cement is therefore predominant.

Formulations where M = 50% show spherical pores, thus confirming the isotropy of the material which was already established by the mechanical tests. The pores appear to be isolated. The distribution of pores is random and homogeneous throughout the material. The sand grains are evenly distributed throughout the material.

The difference in the incorporation of air bubbles in the foam between the C = 18%, w/w_L_ = 2, M = 50% and C = 12%, w/w_L_ = 1.6, M = 50% formulations, which was observed from the density results, is visually confirmed with the tomography images.

Table 5 shows the total porosity (P_tomo_), the maximum radius (R_max_), and the average radius (R_moy_) calculated by the "Pore Size Distribution" plugin from the tomographic data for each formulation.

The difference in the incorporation of air bubbles in the foam between the C = 18% w/w_L_ = 2, M = 50%, and C = 12%, w/w_L_ = 1.6, M = 50% formulations, observed previously in the tomographic images, was also confirmed by the total porosity calculated by the "Pore Size Distribution" plugin from the tomographic data.

The pore size distributions of the C = 12%, w/w_L_ = 1.6, M = 50% and C = 18%, w/w_L_ = 2, M = 50% formulations are different. The C = 18%, w/w_L_ = 2, M = 50% formulation has a maximum pore radius of 1.31 mm, while that of the C = 12%, w/w_L_ = 1.6, M = 50% formulation is equal to 0.75 mm. However, the average radius of the C = 12%, w/w_L_ = 1.6, M = 50% formulation of 0.46 mm is greater than that of the C = 18%, w/w_L_ = 2, M = 50% formulation of 0.29 mm. It would appear that pores specific to the cementitious matrix, such as clay shrinkage and off-gassing, are not responsible for this difference due to their low proportion in relation to the total porosity. There is therefore a difference in the size distribution of the pores produced by the foam, with a lower proportion of small pores for the C = 12%, w/w_L_ = 1.6, M = 50% formulation. This difference is attributed to the low fluidity of the mix for this formulation, which causes small air bubbles to burst upon incorporation.

## 4. Conclusions

Conclusions from this study are summarized as follows:

- The density results, combined with workability, show that it is necessary to have some fluidity in order to introduce the air foam without bursting the air-foam bubbles.
- Obtaining an optimal value of the w/w_L_ ratio for each percentage of cement optimizes the introduction of the air foam and thus optimizes the reduction in density.
- The variation in compressive strength at different curing times indicates that up to 28 days, the increase in compressive strength is lower for the mix design with air foam.
- Compressive strength is significantly impacted by water content. From a w/w_L_ ratio corresponding to the optimization of the introduction of air foam (the value k) in the fresh state, compressive strength is mainly influenced by water content and not air foam.
- With regard to porosity, the average pore radius of the C = 12%, w/w_L_ = 1.6, M = 50% formulation is greater than that of the C = 18%, w/w_L_ = 2, M = 50% formulation (with values, respectively, equal to 0.46 mm and 0.29 mm). This difference can be explained by the low fluidity of the mixture for this formulation, which causes small air bubbles to burst during incorporation.
- There is a balance to be struck between reduced density and compressive strength. Based on the experimental data, a predictive mathematical model of mechanical properties makes it possible to evaluate the density and percentage of the cement with a good reliability.

This study presents promising results for the reuse of fine dredged sediments in foam concrete. For future works, in order to validate the hypotheses presented in this study, other analyses focused on determining the influence of formulation parameters on porosity are currently undergoing testing.

## Figures and Tables

**Table 1:** Characteristics of bentonite.

Swelling	4 mL/g
Specific weight	2.7 g/cm^3^
Apparent density	0.7 g/cm^3^
Sieve non-passing (75 µm) wet sieving	≤4%

**Table 2:** Characteristics of sand.

Petrography	Silica sand
Conformity	Conforming
Granular grade	0/1 mm
Thinness index EN 12620 [31]	0.64
Methylene blue test (0/2) EN 933-9 [32]	0.4 g/kg

**Table 3:** Mechanical and physical characteristics of cement.

Mechanical Performance (MPa)				Time of Initial Setting (min) at 20 °C	Density	Surface Area by Blaine (cm^2^/g)
1 day	2 days	7 days	28 days	145 min	3.17	4250
23	36	55	67			

**Table 4:** Components of cement.

Type	Characteristics (%)	
BUSSAC clinker (K)	CaO/SiO_2_	3.1
	C3S + C2S	78
	MgO	1.0
	Al_2_O_3_	5.1
	C3S	68
	C2S	11
	C3A	6
	C4AF	13

**Table 5:** Characteristics of the pore network calculated by the "Pore Size Distribution" plugin from the tomographic data.

C (%)	w/w_L_	M(%)	P_tomo_ (%)	R_max_ (mm)	R_moy_ (mm)	Standard Deviation (mm)
12	1.6	0	0.9	0.22	0.15	-
		50	7.8	0.75	0.46	0.040
18	2	0	0.6	0.36	0.22	-
		50	25.3	1.31	0.29	0.047

## Data Availability

The original contributions presented in the study are included in the article, further inquiries can be directed to the corresponding author.

## Appendix A

**Table A1:** Results for the fresh state (density and diameter spreading) and the hardened state (mechanical characteristics).

C(%)	w/w_L_	M (%)	Density	Compressive Strength (MPa)	Elastic Modulus (MPa)	Diameter Spreading (mm)
12	1.6	20	1.32	0.77	260	45.33
		30	1.20	0.74	235	37.00
		50	1.24	0.56	206	32.80
	1.8	20	1.26	0.28	239	48.67
		30	1.20	0.23	229	60.00
		50	1.07	0.19	97	60.00
	2	20	1.20	0.15	120	53.00
		30	1.16	-		60.00
		50	1.03	0.15	43	60.00
15	1.6	20	1.41	1.50	522	20.00
		30	1.39	0.58	429	24.00
		50	1.33	0.45	227	24.00
	1.8	20	1.28	0.58	368	48.25
		30	1.21	0.44	292	50.50
		50	1.30	0.33	-	50.50
	2	20	1.23	0.45	168	56.00
		30	1.16	0.38	154	50.00
		50	1.08	0.30	115	51.50
18	1.8	20	1.32	2.30	688	30.50
		30	1.28	1.05	278	35.00
		50	1.20	0.77	238	36.50
	2	20	1.27	0.97	368	35.00
		30	1.19	0.58	231	47.25
		50	1.07	0.47	189	47.25
	2.2	20	1.20	0.52	195	80.00
		30	1.13	0.51	169	58.50
		50	1.05	0.31	117	51.50

## Appendix B

1. Binary image: the grey levels were chosen for each formulation in order to visually select the pores created by the air bubbles in the foam. These correspond to the following:
  - 51 for C = 12%, w/w_L_ = 1.6, M = 0%,
  - 51 for C = 12%, w/w_L_ = 1.6, M = 50%,
  - 50 for C = 18%, w/w_L_ = 2, M = 0%,
  - 62 for C = 18%, w/w_L_ = 2, M = 50%.
2. Process (dilate → dilate → dilate → erode → erode → erode → open → close)
3. Object separation («watershed»)
4. Plugin PSD
